# HER2-low breast cancer shows a lower immune response compared to HER2-negative cases

**DOI:** 10.1038/s41598-022-16898-6

**Published:** 2022-07-28

**Authors:** Nadine S. van den Ende, Marcel Smid, Annemieke Timmermans, Johannes B. van Brakel, Tim Hansum, Renée Foekens, Anita M. A. C. Trapman, Bernadette A. M. Heemskerk-Gerritsen, Agnes Jager, John W. M. Martens, Carolien H. M. van Deurzen

**Affiliations:** 1 Department of Pathology, Erasmus MC Cancer Institute, Erasmus University Medical Centre, ‘s-Gravendijkwal 230, 3015 CE Rotterdam, The Netherlands; 2 Department of Medical Oncology, Erasmus MC Cancer Institute, Erasmus University Medical Centre, Rotterdam, The Netherlands; 3 Department of Pathology, Skåne University Hospital, Malmö, Sweden; 4 Department of Pathology, Reinier Haga MDC, Delft, The Netherlands

**Keywords:** Cancer, Biomarkers, Health care

## Abstract

Currently, the human epidermal growth factor receptor 2 (HER2) status of breast cancer is classified dichotomously as negative or positive to select patients for HER2-targeted therapy. However, with the introduction of novel treatment options, it is important to get more insight in the biology of cancers with low HER2 expression. Therefore, we studied several clinicopathologic characteristics in relation to the level of HER2 expression (HER2- versus HER2low). We used a well-documented cohort of breast cancer patients (n = 529), with available tissue microarrays and Affymetrix mRNA expression data. HER2 status was scored as negative (immunohistochemistry 0) or low (immunohistochemistry 1 + or 2 + without amplification). We associated HER2 status with several clinicopathologic characteristics, gene-expression data and survival, stratified for estrogen receptor (ER) status. Overall, breast cancers were scored as HER2- (n = 429) or HER2low (n = 100). Within the ER+ cohort (n = 305), no significant associations were found between the HER2 groups and clinicopathologic features. However, HER2low tumors showed several differentially expressed genes compared to HER2- cases, including genes that are associated with worse outcome and depletion of immunity. In ER- cases (n = 224), HER2low status was significantly associated with increased regional nodal positivity, lower density of tumor infiltrating lymphocyte and a lower protein expression of Ki-67 and EGFR compared to HER2- cases. After multivariate analysis, only density of tumor infiltrating lymphocytes remained significantly associated with HER2low status (*P* = 0.035). No difference in survival was observed between HER2low and HER2- patients, neither in the ER+ nor ER- cohort. In conclusion, our data suggests that HER2low breast cancer is associated with a lower immune response compared to HER2- breast cancer.

## Introduction

Breast cancer treatment decisions are based, amongst other patient- and tumor characteristics, on the expression of the estrogen receptor (ER), the progesterone receptor (PR) and the human epidermal growth factor receptor 2 (HER2)^1^. HER2 is a protein encoded by the erythroblastic oncogene B (*ERBB2*) gene^2^. Amplification of the oncogene, leading to overexpression of the HER2 protein, plays a role in the development of different breast cancer subtypes by promoting the growth of cancer cells^3^.

In daily clinical practice, the HER2 status of breast cancer is classified dichotomously as either negative or positive to select patients for HER2-targeted therapy. This is usually determined via immunohistochemistry (IHC) and in situ hybridization (ISH)^4^. IHC protein expression is classified as negative (0), weak or partial (1 +), moderate (2 +) or strongly positive (3 +) according to international guidelines^4^. Cases with negative (0) or weak expression (1 +) are considered HER2-negative (HER2-) and patients with strong expression (3 +) are considered as HER2-positive (HER2+). Cases with a moderate protein expression (2 +) need an additional reflex test, like an ISH assay to differentiate between HER2- (ISH without amplification) or HER2+ (ISH with amplification).

Currently, HER2+ patients are eligible for targeted treatment against the HER2 receptor, while patients without HER2 amplification will not receive HER2 blockade treatment^5^. With the introduction of novel HER2-targeting agents in recent years, including antibody–drug conjugates, the clinical relevance of the HER2 classification system is shifting, since patients with low levels of HER2 expression (HER2low) could also have a therapeutic benefit from these agents^3,6^. Antibody–drug conjugates are delivered inside cancer cells by targeting the few HER2 receptors on the cells^6,7^. An advantage of these drugs is that there is a high antibody–drug ratio, thus multiple cytotoxic agents are bound to one antibody molecule^8^. The payload of these drugs is membrane permeable, making it possible to trigger the release of the cytotoxic agent and killing adjacent cells that do not express the HER2 receptor via the bystander effect^6,7,9^. A phase Ib study by Modi et al. showed that 37% of the patients with HER2low metastatic breast cancer had a partial response after treatment with trastuzumab-deruxtecan^10^.

The HER2low category represents tumors with an IHC score of 1 + or 2 + without amplification. According to this definition, HER2- only includes those patients with an IHC score of 0. In HER2low breast cancer cases, the number of receptors is low compared to cases with HER2 amplification^11^. Overall, it is estimated that around 55% of all breast cancers is HER2low^5,7^. Hence, it is important to gain more insight in the biology of cancers with low HER2 expression since this subgroup might be of clinical relevance.

Since HER2low is a relatively new term, data with respect to the clinicopathologic characteristics and the prognostic impact of HER2low breast cancer is limited. Previous research indicated that HER2low tumors are more often ER+ and that they tend to have a higher histologic grade and a higher proliferation rate compared to HER2- tumors^7,12,13^. Study results on the prognostic impact are thus far inconsistent^7,14,15^.

To further understand the biology of the HER2low group, various biomarkers need to be analyzed. So far, an analysis of a PAM50 assay (including 50 breast cancer-related genes) of 3600 patients by Schettini et al. elucidated that hormone receptor positive/HER2low tumors had a higher *ERBB2* expression level than HER2- tumors^5^. Several studies showed that stromal tumor infiltrating lymphocytes (TILs) have a prognostic and predictive value in breast cancer, where a higher density of TILs is correlated with a better outcome^16–18^. High numbers of TILs are associated with triple negative and HER2+ breast cancer. However, it is unknown whether there is a difference in the density of TILs between HER2- and HER2low breast cancer.

Therefore, the aim of this study was to analyze whether there is a relation between several clinicopathologic characteristics, including TILs, a large gene expression dataset and HER2 expression (HER2- versus HER2low), stratified for ER status.

## Results

### General patient and tumor characteristics

From the 720 tumor samples within the cohort, cases with missing (invasive tumor) tissue or missing hormone receptor data (n = 101) were excluded. Furthermore, HER2+ samples (n = 90) were excluded resulting in a final dataset of 529 samples with either HER2- or HER2low breast cancer. Several patient and tumor characteristics of these 529 patients were analyzed and compared between the HER2- and the HER2low cancers (Table 1). Overall, this cohort included 305 patients with ER+ tumors (58%) and 224 with ER- tumors (42%). Most tumors were HER2- (n = 429, 81%), the remaining 100 tumors (19%) were HER2low. In total, 98 patients received adjuvant chemotherapy (e.g., anthracyclines, or anthracycline-containing therapy) and 66 patients received adjuvant hormonal therapy (e.g., tamoxifen, LHRH/tamoxifen) according to historical procedures in the Netherlands.

**Table 1:** Baseline clinicopathologic characteristics of patients with HER2- versus HER2low breast cancer.

Characteristic	ER+ (n = 305, 58%)		*P*-value^a^ univariate	ER- (n = 224, 42%)		*P*-value^a^ univariate	*P*-value multivariate
	HER2- (n = 238, 78%)	HER2low (n = 67, 22%)		HER2- (n = 191, 85%)	HER2low (n = 33, 15%)		
**Age**							
Median in yr (range)	52 (23–88)	55 (32–81)	0.370^b^	52 (22–89)	53 (37–76)	0.387^b^	
Age ≤ 50	99 (42%)	24 (36%)	0.320^c^	86 (45%)	13 (39%)	0.476^c^	
Age > 50	117 (49%)	38 (57%)		90 (47%)	18 (55%)		
Unknown	22 (9%)	5 (7%)		15 (8%)	2 (6%)		
**Menopausal stage**			0.652^c^			0.375^c^	
Premenopausal	101 (42.5%)	31 (46%)		89 (47%)	13 (39%)		
Postmenopausal	115 (48.5%)	31 (46%)		87 (45%)	18 (55%)		
Unknown	22 (9%)	5 (8%)		15 (8%)	2 (6%)		
**Tumor size**			0.777^d^			0.106^c^	
≤ 2 cm (T1)	82 (34.5%)	28 (42%)		58 (30%)	6 (18%)		
> 2 — ≤ 5 cm (T2)	115 (48%)	31 (46%)		103 (54%)	19 (58%)		
> 5 cm (T3)	9 (4%)	2 (2.5%)		8 (4%)	5 (15%)		
(T4)	6 (2.5%)	1 (1.5%)		5 (3%)	1 (3%)		
Unknown (Tx)	26 (11%)	5 (8%)		17 (9%)	2 (6%)		
**Nodal status**			0.440^d^			0.005^d^	0.375
Node negative	140 (59%)	43 (64%)		124 (65%)	14 (42%)		
Node positive	73 (31%)	19 (28%)		51 (27%)	17 (51%)		
Unknown	41 (10%)	5 (8%)		16 (8%)	2 (6%)		
**Distant metastasis status**			0.307^d^			0.580^d^	
Metastasis negative	208 (87.5%)	62 (92.5%)		170 (89%)	31 (94%)		
Metastasis positive	8 (3.5%)	0 (0%)		6 (3%)	0 (0%)		
Unknown	22 (9%)	5 (7.5%)		15 (8%)	2 (6%)		
**Histologic subtype**			0.295^d^			0.134^d^	
Ductal	183 (77%)	54 (80.5%)		153 (80%)	25 (76%)		
Lobular	15 (6%)	1 (1.5%)		11 (6%)	3 (9%)		
Other	38 (16%)	10 (15%)		25 (13%)	3 (9%)		
Unknown	2 (1%)	2 (3%)		2 (1%)	2 (6%)		
**Histologic grade**			0.851^d^			0.661^d^	
1	53 (22%)	14 (21%)		21 (11%)	3 (9%)		
2	89 (37%)	27 (40%)		52 (27%)	11 (33%)		
3	94 (40%)	24 (36%)		116 (61%)	17 (52%)		
Unknown	2 (1%)	2 (3%)		2 (1%)	2 (6%)		
**Mitotic activity count**			0.814^b^			0.327^b^	
Median (range)	8 (0–94)	8 (0–49)		16 (0–112)	15 (0–67)		
**Stromal TIL percentage**			0.077^d^			0.034^d^	0.035
Low (≤ 10)	168 (71%)	52 (78%)		121 (63%)	27 (82%)		
Intermediate (11 — 60)	45 (19%)	5 (7.5%)		38 (20%)	2 (6%)		
High (> 60)	8 (3%)	1 (1.5%)		5 (3%)	3 (9%)		
Unknown	17 (7%)	9 (13%)		27 (14%)	1 (3%)		
**Progesterone receptor**			0.755^d^			0.666^d^	
Negative	56 (23.5%)	17 (25%)		182 (95%)	32 (97%)		
Positive	182 (76.5%)	50 (75%)		9 (5%)	1 (3%)		
**Ki-67 (IHC)**			0.655^d^			0.031^d^	0.145
< 10%	92 (39%)	25 (37%)		63 (33%)	13 (39.5%)		
11 – 25%	47 (19.5%)	16 (24%)		43 (22%)	11 (33.5%)		
> 26%	56 (23.5%)	13 (19.5%)		51 (27%)	2 (6%)		
Unknown	43 (18%)	13 (19.5%)		34 (18%)	7 (21%)		
**EGFR (IHC)**			0.207^d^			0.009^d^	0.402
< 1%	202 (85%)	61 (91%)		136 (71%)	31 (94%)		
> 1%	31 (13%)	5 (7.5%)		51 (27%)	2 (6%)		
Unknown	5 (2%)	1 (1.5%)		4 (2%)	0 (0%)		
**HER2 copy numbers (SISH)**			0.001^d^			< 0.001^d^	
0 — ≤ 2	168 (70.5%)	39 (58%)		125 (65%)	16 (48.5%)		
> 2 — < 6	33 (14%)	22 (33%)		20 (11%)	13 (45.5%)		
Unknown	37 (15.5%)	6 (9%)		46 (24%)	2 (6%)		
**Vascular invasion**			0.089^d^			0.220^d^	
No	178 (75%)	41 (61%)		141 (74%)	20 (61%)		
Yes	57 (24%)	22 (33%)		47 (24.5%)	11 (33%)		
Unknown	3 (1%)	4 (6%)		3 (2%)	2 (6%)		

^a^Unknown values are excluded from analysis.

^b^Mann-Whitney U-test.

^c^Chi-square test.

^d^Fisher’s exact test.

### Clinicopathologic differences between HER2- and HER2low breast cancer

**Figure 1:**
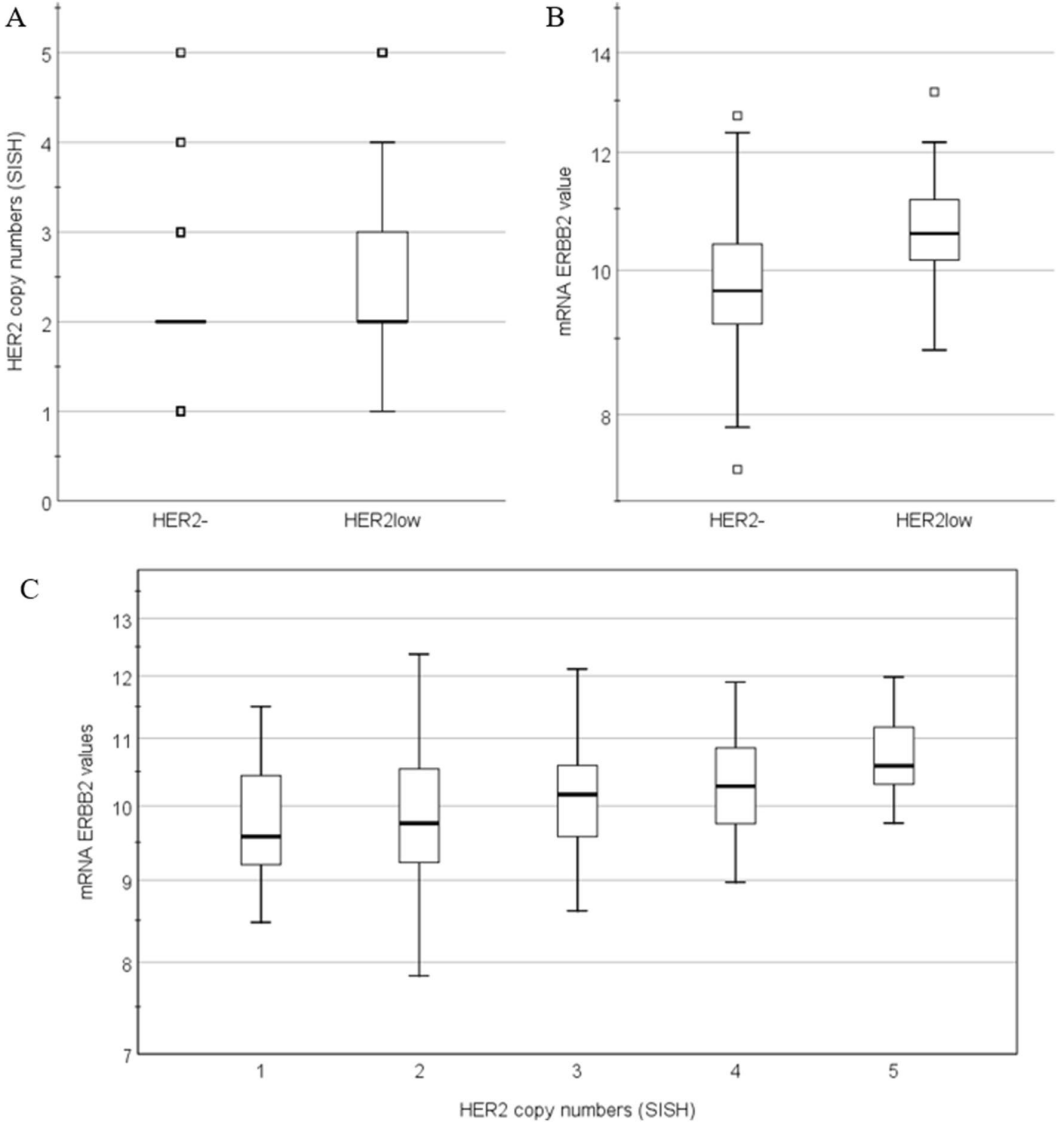
HER2 IHC expression (HER2- versus HER2low) versus HER2 copy number determined by ISH (**A**) and versus mRNA *ERBB2* expression (**B**). Association between the number of HER2 copies and the level of mRNA *ERBB2* expression (**C**). SISH = silver-enhanced in situ hybridization.

The median number of HER2 copies (n = 2), as determined by SISH, was similar in both groups. However, HER2low tumors had a significantly higher HER2 copy number compared to HER2- tumors (*P* ≤ 0.001;  Fig. 1A), in both ER+ and ER- tumors. In the ER+ cohort, there was no significant association between any of the clinicopathologic features between HER2- and HER2low breast cancer, except for the HER2 copy numbers. The level of ER expression by immunohistochemistry (% of positive tumor cells) was not different between ER+HER2- and ER+HER2low cases (*P* = 0.54; t-test).

Within the ER- cohort, HER2low breast cancer was significantly associated with increased regional nodal positivity, lower density of TILs and a lower expression of Ki-67 and epidermal growth factor receptor (EGFR) compared to HER2- cases (*P* < 0.001, *P* = 0.034, *P* = 0.031 and *P* = 0.046 respectively; Table 1). To analyze which of these four characteristics are independently associated with the HER2 status, a multivariate logistic regression analysis was performed for the ER- cohort. After multivariate analysis, only the density of TILs remained significantly associated with HER2low status (*P* = 0.035).

### Gene expression differences between HER2- and HER2low breast cancer

**Figure 2:**
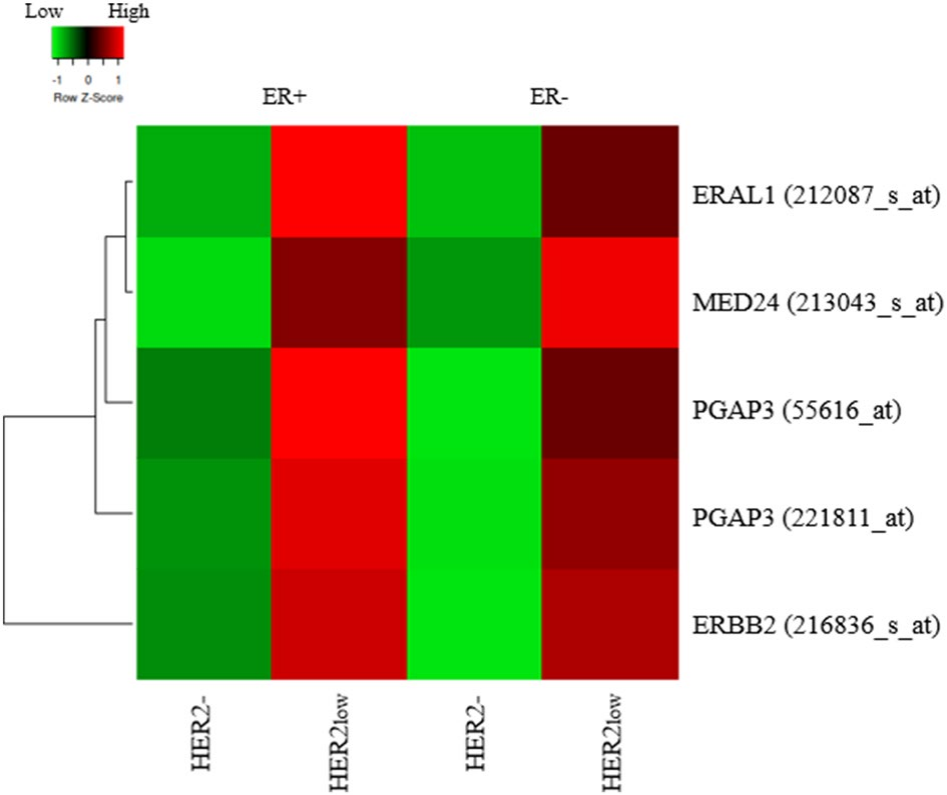
Heat map of genes with a significant different expression level according to the HER2 protein expression (HER2- versus HER2low) and ER status. The red color represents a relatively high level of gene-expression where a green color represents a relatively low level of gene-expression. It is shown that all genes have a higher median expression level in the HER2low cohort compared to the HER2- cohort. This figure was obtained with the use of the heatmapper expression tool from http://www.heatmapper.ca/expression/.

Overall, HER2low cases had a higher mRNA expression of *ERBB2* compared to HER2- cases (*P* < 0.001; Fig. 1B). There was no significant difference in expression of ER-pathway-related genes, neither in the ER+ cohort (ER+HER2- versus ER+HER2low) nor the ER- cohort (ER-HER2- versus ER-HER2low). From the 5000 most variably expressed genes, five probe-sets (4 unique genes) showed significantly higher gene-expression levels (FDR *P* < 0.05) in the HER2low group compared to the HER2- group, within the ER+ cohort (Fig. 2). The four genes were *ERBB2*, Era Like 12S Mitochondrial RRNA Chaperone 1 (*ERAL1*), Mediator Complex Subunit 24 (*MED24*) and Post-GPI Attachment to Proteins Phospholipase 3 (*PGAP3*) genes. The higher expression level effect was also visually seen within the ER- cohort, although none of these four genes showed a statistically significant difference in expression between HER2- and HER2low cancers.

Interdependence of these genes was analyzed by assessing their chromosomal location. Both *MED24* and *PGAP3* are located on the same amplicon as *ERBB2*. *PGAP3* (chr17:39,671,122–39,688,057) is located directly 3’ from *ERBB2* (chr17:39,700,064–39,728,658) and they are both located on the 17q12 cytogenetic band. *MED24* (chr17:40,019,104–40,054,408) is located around 400 kb 5’ from *ERBB2* on cytogenetic band 17q21.1. *ERAL1* (chr17:28,855,016–28,861,061) is located on cytogenetic band 17q11.2, 3’ of *ERBB2*. Furthermore, the Pearson correlation coefficient was calculated to evaluate the relation between the *ERBB2* gene and the other genes. The *ERBB2*/*ERAL1* correlation was 0.370 for the ER+ cases and 0.294 for ER- cases. The correlation between *ERBB2* and *MED24* was 0.464 and 0.260, for ER+ and ER- respectively. For *PGAP3*, two significant probe-sets were found and the correlation was 0.606 (ER+) and 0.552 (ER-) for *ERBB2*/*PGAP3*(55616_at) and 0.688 (ER+) and 0.687 (ER-) for *ERBB2*/*PGAP3*(221811_at). Additionally, it was shown that there is a trend that the level of *ERBB2* expression increased when more HER2 copies (determined by SISH) were present (*P* < 0.001; Fig. 1C). However, no statistical significance was found between the mRNA *ERBB2* expression levels and the HER2 copy numbers (*P* = 0.573).

### Functional pathway enrichment in HER2- and HER2low breast cancer

To gain more insight in the biological difference between HER2- and HER2low breast cancer, a more global gene-expression analysis was performed. For this analysis, all differentially expressed genes with an uncorrected univariate p-value below 0.05 were collected and analyzed for enriched shared biology. In total, 1197 genes for the ER+ cohort and 977 genes for the ER- cohort differentiated significantly between the HER2 groups. Functional annotation clustering of the significant genes within the ER+ cohort revealed an immune related cluster with an enrichment score of 10.93. Within this cluster, a gene ontology biological process pathway was found related to the adaptive immune response (*P* = 6.8E-10) and involved 31 genes (Supplementary Table 1). Furthermore, another immune related gene ontology biological process pathway was detected, independent of the immune cluster. This pathway was presented with the name immune response and involved 69 genes (*P* = 1.5E-15; Supplementary Table 2). The expression levels of these genes were higher in the HER2- group compared to the HER2low group. Some potential interesting genes retrieved from these pathways are known to contribute to an increased immunity, for example by regulating T-cell activation or by improving T-cell proliferation or helping with T-cell mediated killing. Within the ER- cohort, no enriched immunity pathways were detected.

### Survival data of patients with HER2- versus HER2low breast cancer

**Figure 3:**
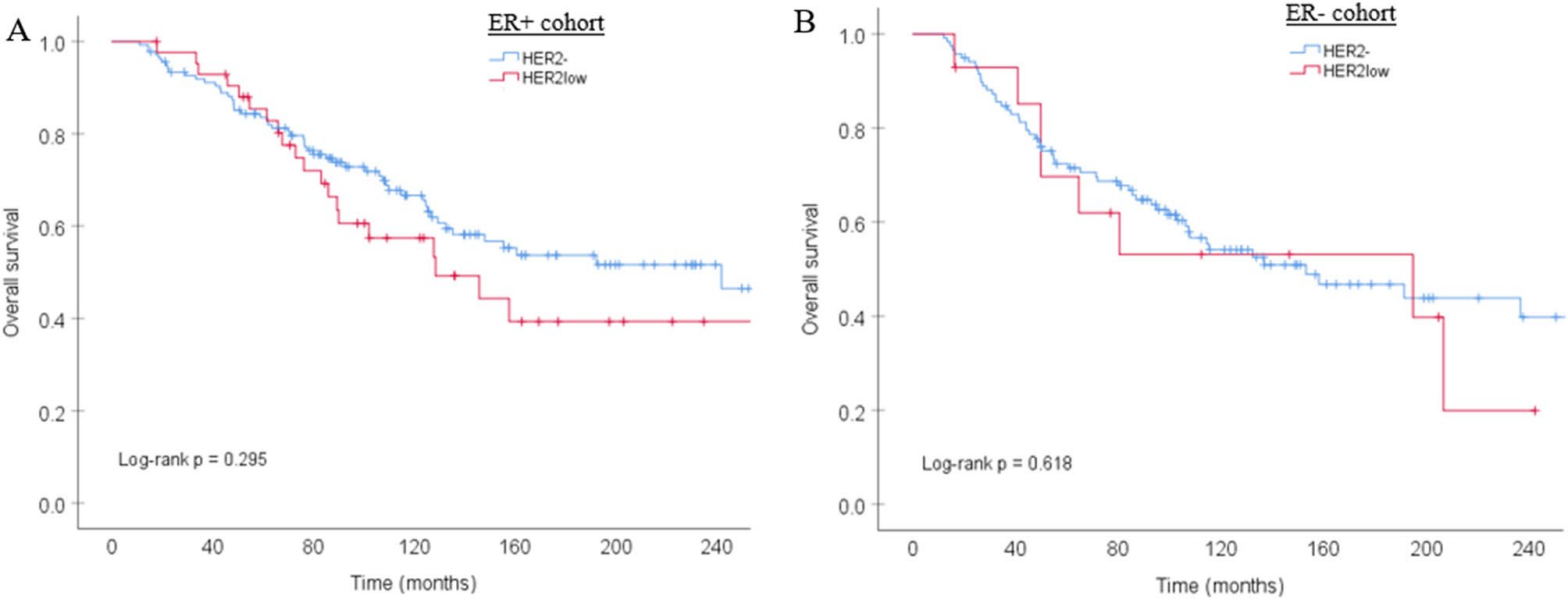
Kaplan Meier curves of overall survival according to HER2 status within the ER+ cohort (**A**) and the ER- cohort (**B**).

From the data set of 529 patients, additional patients were excluded based on missing clinical data (n = 44), receiving adjuvant systemic therapy (n = 115), positive nodal or distant metastasis status at diagnosis (n = 52), leading to a survival analysis of 318 patients. Median follow-up was 82 months for the ER+ cases and 64 months for the ER- cases. Regarding overall survival, the Kaplan–Meier survival curves were not significantly different for patients with HER2- and HER2low breast cancer, neither within the ER+ cohort nor in the ER- cohort (*P* = 0.295 and- *P* = 0.618 respectively; Fig. 3). Furthermore, there were no differences regarding disease free survival (*P* = 0.664 for ER+ cases and *P* = 0.391 for ER- cases) and metastasis free survival (*P* = 0.615 for ER+ cases and *P* = 0.941 for ER- cases) between patients with HER2- and HER2low breast cancer (Kaplan–Meier curves not shown).

## Discussion

With the introduction of novel antibody drug conjugates, which can also target tumors with low levels of HER2 expression, there is a need for a more granular HER2 classification system instead of a dichotomous division in either negative or positive. We aimed to analyze whether HER2low primary breast cancers are different compared to HER2- tumors with respect to clinicopathologic characteristics, gene-expression and survival. In this study, we excluded HER2+ cases since this breast cancer subtype is already known to have a distinct biology.

Overall, most breast cancers were scored as HER2- in our study (n = 429). The remaining patients (n = 100) were HER2low. This finding is in contrast with previous literature, reporting that around 55% of all breast cancers is HER2low^3,7^. This difference could be explained by the relatively large proportion of ER- cases in our study and/or the use of breast cancer tissue from a historical cohort.

HER2low breast cancers showed a significant higher number of HER2 copies and a higher *ERBB2* gene-expression compared to HER2- tumors, which is in line with previous studies^5,15^. Within the ER+ cohort (n = 305), gene-expression analyses showed that HER2low tumors showed several differentially expressed genes compared to HER2- cases. This included *ERBB2*, *ERAL1*, *MED24* and *PGAP3*, which were all higher expressed in the HER2low group. Based on genomic co-localization and the correlation coefficients of *ERBB2*, *MED24* and *PGAP3*, the higher expression levels are likely the result of amplification of a common chromosomal region. For *ERAL1*, expression levels show little support for this co-amplification indicating that it is likely regulated otherwise. Previous research has shown that most of these genes are linked to worse prognosis for breast cancer patients. *MED24* has been reported to have a function in the growth of breast cancer cells^19^. *PGAP3* was identified as a promotor of growth and metastasis in triple negative breast cancer^20^. *ERAL1* is a mitochondrial RNA chaperone which has not been associated with breast cancer prognosis before^21^. However, *ERAL1* is involved in the formation of the 28S small mitochondrial ribosomal protein (*MRPS28*) and that protein has been shown to be involved with breast cancer proliferation and metastasis^22^. In this ER+ cohort, pathway analyses additionally showed enrichment of immune-related genes in the HER2- group compared to the HER2low group.

In ER- cases (n = 224), HER2low status was significantly associated with increased regional nodal positivity, lower density of TILs and a lower protein expression of Ki-67 and EGFR compared to HER2- cases. After multivariate analysis, only density of TILs remained significantly associated with HER2low status. This suggests that ER-/HER2- tumors have a more basal-like profile compared to ER-/HER2low tumors^23,24^. In line with the ER+ cohort, gene-expression analyses of the ER- cohort also showed a trend toward higher expression of *ERAL1*, *MED24* and *PGAP3* for the HER2low cases, although this was not significant. No enriched immunity pathways were detected within the ER- cohort.

Literature regarding HER2low in relation to gene-expression is very scarce. Schettini et al. analyzed a set of 55 genes of which 34 showed a significant difference between HER2low and HER2- breast cancer within the ER+ cohort^5^. In our study, no genes (after correcting for multiple testing) were found to be statistically significant within the ER- cohort, which is in line with Schettini et al. who also did not find any significant differences in genes within the ER- group. Furthermore, HER2- tumors were more enriched in immune-related genes, which is concordant with the study of Schettini et al. reporting that HER2- tumors are more basal-like, using the PAM50 assay, than HER2low tumors. The higher expression of EGFR in ER-HER2- tumors compared to ER-HER2low tumors in the univariate analysis of our study also supports a more basal-like TNBC aspect of HER2- cases^25,26^.

Overall, our results suggest that HER2low breast cancer is associated with a limited immune response compared to HER2- breast cancer, as shown by the gene-expression data of the ER+ cohort and the TIL-score of the ER- cohort. Several previous studies reported that high levels of TILs are associated with a higher probability of treatment response and an improved outcome^18,27,28^. In our study, no difference in survival was observed between these two HER2 groups, neither in the ER+ nor in the ER- cohort. Previous literature is inconsistent with respect to outcome. In line with our findings, various studies reported no difference in overall survival between HER2- and HER2low breast cancer patients^5,15,29^. Denkert et al. reported that ER+/HER2low breast cancers have a lower pathological complete response rate after neoadjuvant chemotherapy compared to ER+/HER2- cancers^14^. Furthermore, they concluded that patients with HER2low breast cancer have a better prognosis compared to HER2- cases, in the ER- cohort. Other previous studies reported that HER2low is associated with worse prognosis, in ER+ breast cancer^7,30^.

This is the first study that analyzes the HER2low status of breast cancer in relation to the density of TILs and a large gene-expression dataset. Furthermore, we used a well-documented cohort of patients. Since ER expression is regarded as a key-factor for tumor biology and outcome, we stratified for ER status^5^. However, this study also has some limitations. First, the dataset was based on a historical multicenter cohort of patients for which gene-expression data was generated for different research questions^31–34^. Although it is a cohort with a long follow-up period, there was a relatively large proportion of dropout, resulting in a relatively short median follow-up time. Especially for the ER+ cohort it is known that longer follow-up time is needed to detect disease recurrence^35^. Besides, this could have influenced the tissue quality and thus the HER2 protein expression levels, as reflected in the relatively low proportion of HER2low cases in our series^36–38^. However, this would rather result in an underestimation of our findings, since the HER2- cases might include some HER2low cases of which the HER2 protein expression levels have decreased slightly. Finally, scoring of HER2 status and density of TILs was performed according to international guidelines, but inter-observer variability has been reported^39–42^. In addition, HER2 expression was scored on TMAs, so heterogeneity of the tumors could not be completely depicted. Future mechanistic studies could elucidate the mechanism of poor immune infiltration into HER2low tumors.

In summary, in the ER+ cohort, we observed that HER2low tumors had a different gene-expression pattern compared to HER2- cancers, including genes that are associated with depletion of immunity. In ER- cases, HER2low cancers had a lower density of TILs compared to HER2- cases. Although immunity is regarded as an important prognostic factor in breast cancer, we did not observe a difference in survival between HER2low and HER2- patients, neither in the ER+ nor in the ER- cohort. Future research based on large, more recent cohorts of patients, could further elucidate the clinical relevance of HER2low in relation to immunity.

## Methods

### General patient and tumor characteristics

This retrospective study was based on a well-documented cohort of primary breast cancer patients, for whom cancer tissues were available on tissue microarrays and Affymetrix data was known. Patients were diagnosed between 1982 and 2003 in multiple centers across the Netherlands. Coded leftover patient material was used in accordance with the Code of Conduct of the Federation of Medical Scientific Societies in the Netherlands^43^. According to these national guidelines, this work was not subject to the Medical Research Involving Human Subjects Act (WMO; METC 02.593).

**Figure 4:**
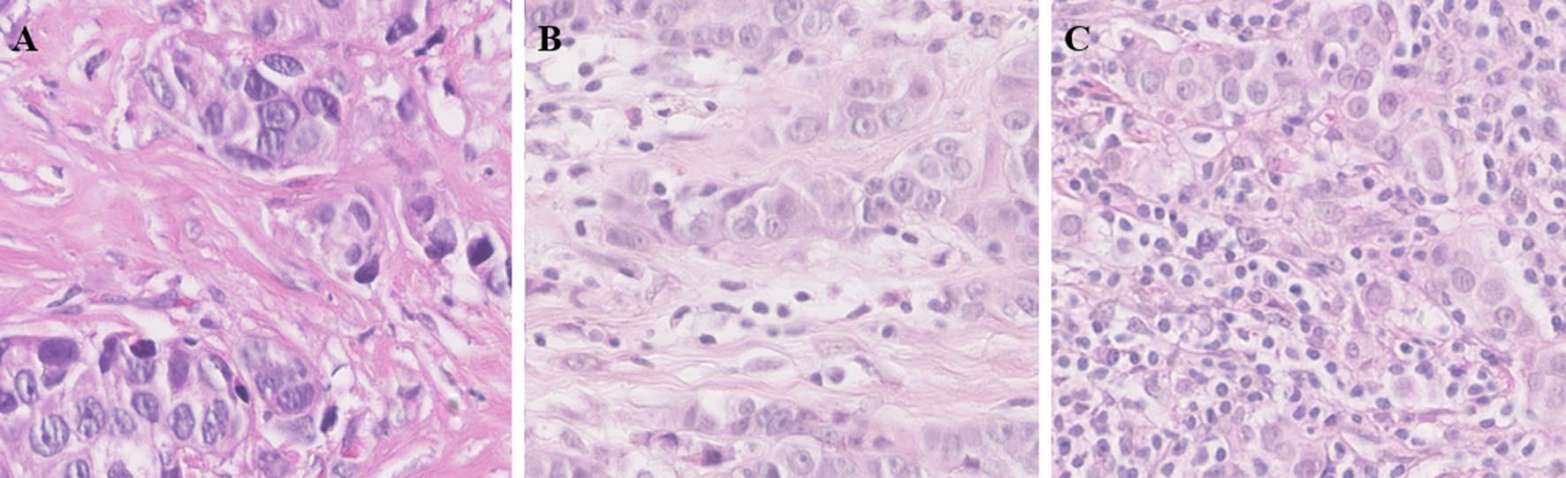
Representative images of breast cancers with a low (**A**), intermediate (**B**) or high (**C**) density of stromal TILs (Hematoxylin and eosin staining at a 60× magnification).

In total, formalin-fixed-paraffin-embedded breast cancer tissue of 720 tumors were analyzed. Clinical data and tumor characteristics were partly collected from medical charts and pathology reports. This included age, menopausal status, pTNM classification, treatment and outcome data (overall survival, disease free survival and metastasis free survival). Central pathology review of whole sections was performed to assess histologic grade, histologic subtype, vascular invasion, mitotic activity index and density of TILs. Histologic grading was determined using the Nottingham modified Bloom and Richardson scoring system^44^. The percentage of stromal TILs was scored on hematoxylin and eosin-stained whole slides according to the recommendations of the International TILs Working Group^45,46^. Figure 4 illustrates examples of breast cancers with a low, intermediate or high density of TILs.

### Tissue microarray scoring

**Figure 5:**
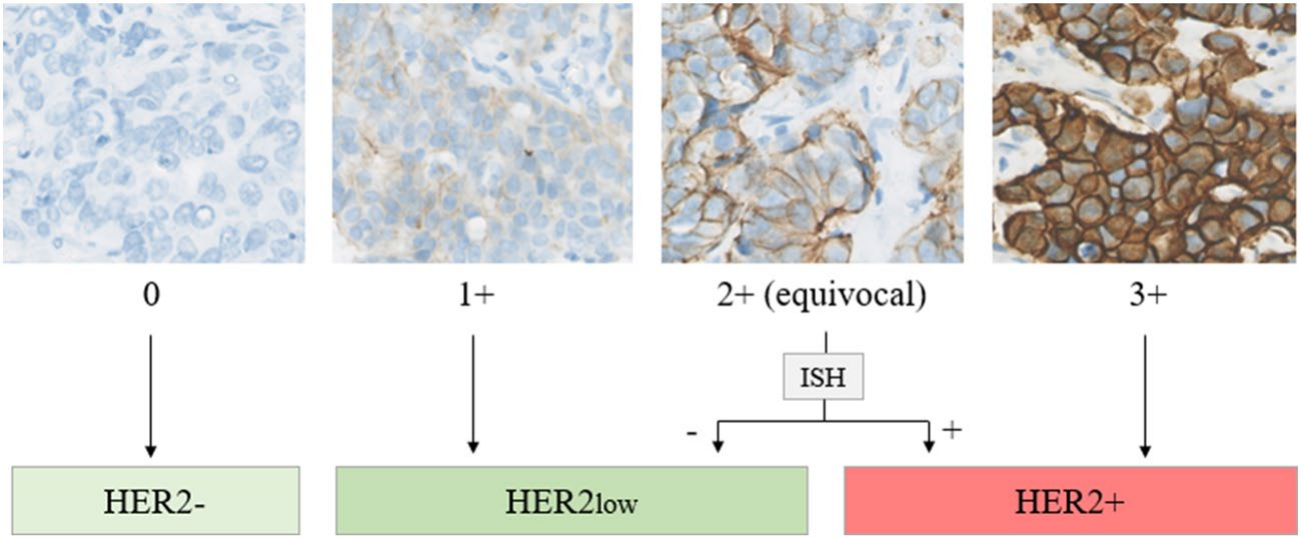
A schematic overview of HER2 scoring in breast cancer, as used in this study. Breast carcinomas are considered HER2- if the IHC score is 0. An IHC score 1 + or 2 + without HER2 amplification (after ISH) is categorized as HER2low. Breast cancers with an IHC score of 2 + with amplification (after ISH) or 3 + are HER2+ (80× magnification). ISH = in situ hybridization.

Breast cancer tissues of all patients was available in triplicate on tissue microarrays. Sections of 4 µm were cut (Micron HM340E) and mounted on Superfrost plus slides (Menzel-Glaser, Braunschweig, Germany). Protein expression of ER, PR, HER2, Ki-67 and EGFR on invasive tumor cells was scored manually by two observers in a central lab^47^. The ER and PR status was reported as negative or positive, using a cut off at 10% stained cells, according to the Dutch treatment guidelines^48^. The Ki-67 expression for this cohort was categorized as low (≤ 10%), intermediate (11–25%) or high (≥ 26%)^49–51^. For this study, tissue microarrays were immunohistochemically stained with the 4B5 anti-HER-2/neu antibody (Ventana BenchMark ULTRA, ROCHE), using cell lines and human tissues as internal controls. The VM NanoZoomer 2.0-HT (Hamamatsu Photonics K.K.) was used to digitize the slides. HER2 status was scored according to the most recent guidelines from The American Society of Clinical Oncology/College of American Pathologists (ASCO/CAP)^4^. HER2- breast carcinomas were defined by an IHC score of 0, whereas HER2low carcinomas were defined by an IHC score of 1 + or 2 + without amplification (Fig. 5). HER2+ was assigned according to international guidelines as IHC 2 + with amplification or IHC 3 +^3^.

HER2 silver-enhanced in situ hybridization (SISH) was performed using the VENTANA HER2 Dual ISH DNA Probe Cocktail assay (Ventana BenchMark ULTRA, ROCHE). A HER2 copy number of < 6 per cell was considered HER2 non-amplified and ≥ 6 copies per cell was considered as HER2-amplified^4^. The triplicate IHC and ISH scores were combined to a final HER2 score, using the core with the highest level of expression in case of discrepancy. Patients without assessable invasive tumor tissue were excluded.

### Statistical analysis

The statistical analysis was performed using IBM SPPS Statistics version 26. The Pearson Chi-square or Fisher’s exact tests were used to investigate differences between HER2- and HER2low cases for the categorical variables, stratified for ER status. For the continuous variables a Mann–Whitney U-test was performed. A linear trend test was performed for the categorical variables with a minimum of 4 categories. Multivariate logistic regression analysis was performed to analyze whether relevant, univariate significant, variables were independently associated with HER2 status. For survival analysis, overall survival, disease free survival and metastasis free survival were used as endpoints. The overall survival was defined as the time from diagnosis to date of death or the last date where the patients were known to be alive. Disease free survival was defined as the time from diagnosis to the date of disease recurrence, last follow-up or death (of any cause). Disease recurrence was defined by a positive biopsy within either the ipsilateral breast or axillary nodes. Metastasis free survival was defined as the time from diagnosis to the date of distant disease recurrence, last follow-up or death. With the use of the Mantel-Cox method, Kaplan–Meier curves of the survival data were visualized. Differences in outcome between the HER2 subgroups were evaluated by Log-rank tests, where a two-sided p-value below 0.05 was considered as statistically significant.

Gene-expression levels were derived from existing in-house data; samples were run on both U133A and HGU133Plus2.0 chips. The samples were previously described and are available via the Gene Expression Omnibus (http://www.ncbi.nlm.nih.gov/geo/) with accession codes GSE2034, GSE5327, GSE12276 and GSE27830^31–34^. Raw data were normalized using fRMA and samples from both platforms were combined using probe-sets common to both chip-types^52^. ComBat was used to correct for batch effects resulting from using data of two different platforms^53^.

Next, the top 5000 most variable (highest standard deviation) genes were used for further analysis. Differentially expressed genes were identified using the non-parametric Mann–Whitney U-test in STATA v14 (StataCorp, Houston, USA). Chromosome locations of the genes were retrieved via http://genome.ucsc.edu and cytogenetic band locations were retrieved from www.genecards.org. An overview of the expression of the genes is presented with the use of heatmapper.ca expression tool. Furthermore, a functional pathway analysis was performed using DAVID bioinformatics resources 6.8, to investigate the global role of differentially expressed genes for HER2low breast cancer and analyzed for enriched shared biology^54,55^.

### Ethics approval and consent to participate

This work was approved and need of informed consent was waived by the Medical Ethics Committee of the Erasmus MC (MEC 02.953). This Medical Ethics Committee of the Erasmus MC approved that the rules laid down in the Medical Research Involving Human Subjects Act do not apply to this work. Therefore, there was no need for an informed consent. The study was performed in accordance with the Declaration of Helsinki.

## Supplementary Information

Supplementary Information.

## Data Availability

The datasets used and analyzed during this study are available by Corresponding author on reasonable request.

## Abbreviations

ER: Estrogen receptor
ERBB2: Erythroblastic oncogene B
HER2: Human epidermal growth factor 2
IHC: Immunohistochemistry
ISH: in situ Hybridization
(s)TIL: (Stromal) tumor infiltrating lymphocyte
