# Supplementary material for: HER2-low breast cancer shows a lower immune response compared to HER2-negative cases
Source: Sci Rep. 2022 Jul 28;12:12974. doi: 10.1038/s41598-022-16898-6 (PMC9334272; doi:10.1038/s41598-022-16898-6)
Supplement: Supplementary file 1 — Supplementary Information. [file 41598_2022_16898_MOESM1_ESM.docx]

**Supplementary data**

HER2-low breast cancer shows a lower immune response compared to HER2-negative cases

Nadine S. van den Ende^1^, Marcel Smid^2^, Annemieke Timmermans^2^, Johannes B. van Brakel^1,3^, Tim Hansum^1,4^, Renée Foekens^2^, Anita M.A.C. Trapman^2^, Bernadette A.M. Heemskerk-Gerritsen^2^, Agnes Jager^2^, John W.M. Martens^2^, Carolien H.M. van Deurzen^1,^*

^1^ Department of Pathology, Erasmus MC Cancer Institute, Erasmus University Medical Centre, Rotterdam, The Netherlands

^2^ Department of Medical Oncology, Erasmus MC Cancer Institute, Erasmus University Medical Centre, Rotterdam, The Netherlands

^3^ Department of Pathology, Skåne University Hospital, Malmö, Sweden

^4^ Department of Pathology, Reinier Haga MDC, Delft, The Netherlands

**Supplementary table 1.**

Genes involved in the significantly enriched adaptive immune response pathway (p=6.8e-10).

**Supplementary table 2.**

Genes involved in the significantly enriched immune response pathway (p=1.5e-15).

**Supplementary table 1.**

| **ID** | **Gene Name** | **Species** |
| --- | --- | --- |
| 206682_at | C-type lectin domain family 10 member A(CLEC10A) | Homo sapiens |
| 215784_at | CD1e molecule(CD1E) | Homo sapiens |
| 203547_at | CD4 molecule(CD4) | Homo sapiens |
| 205049_s_at | CD79a molecule(CD79A) | Homo sapiens |
| 205988_at | CD84 molecule(CD84) | Homo sapiens |
| 210895_s_at | CD86 molecule(CD86) | Homo sapiens |
| 222233_s_at | DNA cross-link repair 1C(DCLRE1C) | Homo sapiens |
| 212486_s_at; 210105_s_at; 216033_s_at | FYN proto-oncogene, Src family tyrosine kinase(FYN) | Homo sapiens |
| 205419_at | G protein-coupled receptor 183(GPR183) | Homo sapiens |
| 211339_s_at | IL2 inducible T-cell kinase(ITK) | Homo sapiens |
| 205842_s_at | Janus kinase 2(JAK2) | Homo sapiens |
| 205821_at | KLRC4-KLRK1 readthrough(KLRC4-KLRK1) | Homo sapiens |
| 202625_at; 210754_s_at; 202626_s_at | LYN proto-oncogene, Src family tyrosine kinase(LYN) | Homo sapiens |
| 210116_at | SH2 domain containing 1A(SH2D1A) | Homo sapiens |
| 217147_s_at | T cell receptor associated transmembrane adaptor 1(TRAT1) | Homo sapiens |
| 206641_at | TNF receptor superfamily member 17(TNFRSF17) | Homo sapiens |
| 218856_at; 214581_x_at | TNF receptor superfamily member 21(TNFRSF21) | Homo sapiens |
| 201952_at | activated leukocyte cell adhesion molecule(ALCAM) | Homo sapiens |
| 201012_at | annexin A1(ANXA1) | Homo sapiens |
| 202902_s_at | cathepsin S(CTSS) | Homo sapiens |
| 206914_at | cytotoxic and regulatory T-cell molecule(CRTAM) | Homo sapiens |
| 204988_at | fibrinogen beta chain(FGB) | Homo sapiens |
| 216491_x_at; 211634_x_at; 209374_s_at; 215949_x_at | immunoglobulin heavy constant mu(IGHM) | Homo sapiens |
| 210354_at | interferon gamma(IFNG) | Homo sapiens |
| 212592_at | joining chain of multimeric IgA and IgM(JCHAIN) | Homo sapiens |
| 210152_at | leukocyte immunoglobulin like receptor B4(LILRB4) | Homo sapiens |
| 207734_at | lymphocyte transmembrane adaptor 1(LAX1) | Homo sapiens |
| 205569_at | lysosomal associated membrane protein 3(LAMP3) | Homo sapiens |
| 200904_at | major histocompatibility complex; class I; E(HLA-E) | Homo sapiens |
| 203879_at | phosphatidylinositol-4,5-bisphosphate 3-kinase catalytic subunit delta(PIK3CD) | Homo sapiens |
| 207957_s_at; 209685_s_at | protein kinase C beta(PRKCB) | Homo sapiens |

**Supplementary table 2.**

| **ID** | **Gene Name** | **Species** |
| --- | --- | --- |
| 202869_at | 2'-5'-oligoadenylate synthetase 1(OAS1) | Homo sapiens |
| 32128_at | C-C motif chemokine ligand 18(CCL18) | Homo sapiens |
| 210072_at | C-C motif chemokine ligand 19(CCL19) | Homo sapiens |
| 204103_at | C-C motif chemokine ligand 4(CCL4) | Homo sapiens |
| 204655_at, 1405_i_at | C-C motif chemokine ligand 5(CCL5) | Homo sapiens |
| 206978_at | C-C motif chemokine receptor 2(CCR2) | Homo sapiens |
| 206991_s_at | C-C motif chemokine receptor 5 (gene/pseudogene)(CCR5) | Homo sapiens |
| 206337_at | C-C motif chemokine receptor 7(CCR7) | Homo sapiens |
| 204470_at | C-X-C motif chemokine ligand 1(CXCL1) | Homo sapiens |
| 203915_at | C-X-C motif chemokine ligand 9(CXCL9) | Homo sapiens |
| 823_at | C-X3-C motif chemokine ligand 1(CX3CL1) | Homo sapiens |
| 215784_at | CD1e molecule(CD1E) | Homo sapiens |
| 206150_at | CD27 molecule(CD27) | Homo sapiens |
| 203547_at | CD4 molecule(CD4) | Homo sapiens |
| 209619_at | CD74 molecule(CD74) | Homo sapiens |
| 210895_s_at | CD86 molecule(CD86) | Homo sapiens |
| 205758_at | CD8a molecule(CD8A) | Homo sapiens |
| 204780_s_at, 215719_x_at, 216252_x_at, 204781_s_at | Fas cell surface death receptor(FAS) | Homo sapiens |
| 210889_s_at | Fc fragment of IgG receptor IIb(FCGR2B) | Homo sapiens |
| 205419_at | G protein-coupled receptor 183(GPR183) | Homo sapiens |
| 211796_s_at, 213193_x_at, 210915_x_at | T cell receptor beta constant 1(TRBC1) | Homo sapiens |
| 218856_at, 214581_x_at | TNF receptor superfamily member 21(TNFRSF21) | Homo sapiens |
| 207536_s_at | TNF receptor superfamily member 9(TNFRSF9) | Homo sapiens |
| 202910_s_at | adhesion G protein-coupled receptor E5(ADGRE5) | Homo sapiens |
| 201487_at | cathepsin C(CTSC) | Homo sapiens |
| 202902_s_at | cathepsin S(CTSS) | Homo sapiens |
| 212067_s_at | complement C1r(C1R) | Homo sapiens |
| 217767_at | complement C3(C3) | Homo sapiens |
| 210140_at | cystatin F(CST7) | Homo sapiens |
| 205081_at | cysteine rich protein 1(CRIP1) | Homo sapiens |
| 205066_s_at | ectonucleotide pyrophosphatase/phosphodiesterase 1(ENPP1) | Homo sapiens |
| 210321_at | granzyme H(GZMH) | Homo sapiens |
| 202748_at | guanylate binding protein 2(GBP2) | Homo sapiens |
| 211649_x_at, 216542_x_at | immunoglobulin heavy constant alpha 1(IGHA1) | Homo sapiens |
| 214973_x_at | immunoglobulin heavy constant delta(IGHD) | Homo sapiens |
| 214777_at, 217157_x_at, 215176_x_at, 216576_x_at, 211643_x_at, 211644_x_at, 214836_x_at, 221671_x_at, 214669_x_at, 216829_at, 216207_x_at, 214768_x_at, 221651_x_at | immunoglobulin kappa constant(IGKC) | Homo sapiens |
| 216560_x_at, 215214_at, 209138_x_at, 214677_x_at | immunoglobulin lambda constant 1(IGLC1) | Homo sapiens |
| 215379_x_at, 217258_x_at, 217227_x_at, 216430_x_at | immunoglobulin lambda variable 1-44(IGLV1-44) | Homo sapiens |
| 203627_at | insulin like growth factor 1 receptor(IGF1R) | Homo sapiens |
| 210354_at | interferon gamma(IFNG) | Homo sapiens |
| 204057_at | interferon regulatory factor 8(IRF8) | Homo sapiens |
| 202948_at | interleukin 1 receptor type 1(IL1R1) | Homo sapiens |
| 209827_s_at | interleukin 16(IL16) | Homo sapiens |
| 204116_at | interleukin 2 receptor subunit gamma(IL2RG) | Homo sapiens |
| 205926_at | interleukin 27 receptor subunit alpha(IL27RA) | Homo sapiens |
| 203828_s_at | interleukin 32(IL32) | Homo sapiens |
| 205798_at | interleukin 7 receptor(IL7R) | Homo sapiens |
| 212592_at | joining chain of multimeric IgA and IgM(JCHAIN) | Homo sapiens |
| 205668_at | lymphocyte antigen 75(LY75) | Homo sapiens |
| 205269_at, 205270_s_at | lymphocyte cytosolic protein 2(LCP2) | Homo sapiens |
| 207734_at | lymphocyte transmembrane adaptor 1(LAX1) | Homo sapiens |
| 207339_s_at | lymphotoxin beta(LTB) | Homo sapiens |
| 200904_at | major histocompatibility complex, class I, E(HLA-E) | Homo sapiens |
| 217478_s_at | major histocompatibility complex, class II, DM alpha(HLA-DMA) | Homo sapiens |
| 203932_at | major histocompatibility complex, class II, DM beta(HLA-DMB) | Homo sapiens |
| 205671_s_at | major histocompatibility complex, class II, DO beta(HLA-DOB) | Homo sapiens |
| 211991_s_at | major histocompatibility complex, class II, DP alpha 1(HLA-DPA1) | Homo sapiens |
| 201137_s_at | major histocompatibility complex, class II, DP beta 1(HLA-DPB1) | Homo sapiens |
| 212671_s_at | major histocompatibility complex, class II, DQ alpha 1(HLA-DQA1) | Homo sapiens |
| 209823_x_at, 209312_x_at, 215193_x_at, 211654_x_at, 212998_x_at, 211656_x_at | major histocompatibility complex, class II, DQ beta 1(HLA-DQB1) | Homo sapiens |
| 215536_at | major histocompatibility complex, class II, DQ beta 2(HLA-DQB2) | Homo sapiens |
| 210982_s_at, 208894_at | major histocompatibility complex, class II, DR alpha(HLA-DRA) | Homo sapiens |
| 208306_x_at, 204670_x_at | major histocompatibility complex, class II, DR beta 1(HLA-DRB1) | Homo sapiens |
| 203574_at | nuclear factor, interleukin 3 regulated(NFIL3) | Homo sapiens |
| 204897_at | prostaglandin E receptor 4(PTGER4) | Homo sapiens |
| 203528_at | semaphorin 4D(SEMA4D) | Homo sapiens |
| 210176_at | toll like receptor 1(TLR1) | Homo sapiens |
| 204731_at | transforming growth factor beta receptor 3(TGFBR3) | Homo sapiens |
| 213293_s_at | tripartite motif containing 22(TRIM22) | Homo sapiens |
